# Segregating Complex Sound Sources through Temporal Coherence

**DOI:** 10.1371/journal.pcbi.1003985

**Published:** 2014-12-18

**Authors:** Lakshmi Krishnan, Mounya Elhilali, Shihab Shamma

**Affiliations:** 1 Department of Electrical and Computer Engineering, University of Maryland, College Park, Maryland, United States of America; 2 Department of Electrical and Computer Engineering, The Johns Hopkins University, Baltimore, Maryland, United States of America; 3 Department Etudes Cognitive, Ecole Normale Superieure, Paris, France; Case Western Reserve University, United States of America

## Abstract

A new approach for the segregation of monaural sound mixtures is presented based on the principle of temporal coherence and using auditory cortical representations. Temporal coherence is the notion that perceived sources emit coherently modulated features that evoke highly-coincident neural response patterns. By clustering the feature channels with coincident responses and reconstructing their input, one may segregate the underlying source from the simultaneously interfering signals that are uncorrelated with it. The proposed algorithm requires no prior information or training on the sources. It can, however, gracefully incorporate cognitive functions and influences such as memories of a target source or attention to a specific set of its attributes so as to segregate it from its background. Aside from its unusual structure and computational innovations, the proposed model provides testable hypotheses of the physiological mechanisms of this ubiquitous and remarkable perceptual ability, and of its psychophysical manifestations in navigating complex sensory environments.

## Author Summary

Humans and many animals can effortlessly navigate complex sensory environments, segregating and attending to one desired target source while suppressing distracting and interfering others. In this paper, we present an algorithmic model that can accomplish this task with no prior information or training on complex signals such as speech mixtures, and speech in noise and music. The model accounts for this ability relying solely on the temporal coherence principle, the notion that perceived sources emit coherently modulated features that evoke coincident cortical response patterns. It further demonstrates how basic cortical mechanisms common to all sensory systems can implement the necessary representations, as well as the adaptive computations necessary to maintain continuity by tracking slowly changing characteristics of different sources in a scene.

## Introduction

Humans and animals can attend to a sound source and segregate it rapidly from a background of many other sources, with no learning or prior exposure to the specific sounds. For humans, this is the essence of the well-known *cocktail party problem* in which a person can effortlessly conduct a conversation with a new acquaintance in a crowded and noisy environment [1], [2]. For frogs, songbirds, and penguins, this ability is vital for locating a mate or an offspring in the midst of a loud chorus [3], [4]. This capacity is matched by comparable object segregation feats in vision and other senses [5], [6], and hence understanding it will shed light on the neural mechanisms that are fundamental and ubiquitous across all sensory systems.

Computational models of auditory scene analysis have been proposed in the past to disentangle source mixtures and hence capture the functionality of this perceptual process. The models differ substantially in flavor and complexity depending on their overall objectives. For instance, some rely on prior information to segregate a specific target source or voice, and are usually able to reconstruct it with excellent quality [7]. Another class of algorithms relies on the availability of multiple microphones and the statistical independence among the sources to separate them, using for example ICA approaches or beam-forming principles [8]. Others are constrained by a single microphone and have instead opted to compute the spectrogram of the mixture, and then to decompose it into separate sources relying on heuristics, training, mild constraints on matrix factorizations [9]–[11], spectrotemporal masks [12], and gestalt rules [1], [13], [14]. A different class of approaches emphasizes the biological mechanisms underlying this process, and assesses both their plausibility and ability to replicate faithfully the psychoacoustics of stream segregation (with all their strengths and weaknesses). Examples of the latter approaches include models of the auditory periphery that explain how simple tone sequences may stream [15]–[17], how pitch modulations can be extracted and used to segregate sources of different pitch [18]–[20], and models that handle more elaborate sound sequences and bistable perceptual phenomena [10], [21]–[23]. Finally, of particular relevance here are algorithms that rely on the notion that features extracted from a given sound source can be bound together by correlations of intrinsic coupled oscillators in neural networks that form their connectivity online [23], [24]. It is fair to say, however, that the diversity of approaches and the continued strong interest in this problem suggest that no algorithm has yet achieved sufficient success to render the “cocktail party problem" solved from a theoretical, physiological, or applications point of view.

While our approach echoes some of the implicit or explicit ideas in the above-mentioned algorithms, it differs fundamentally in its overall framework and implementation. It is based on the notion that perceived sources (sound streams or objects) emit features, that are modulated in strength in a largely temporally coherent manner and that they evoke highly correlated response patterns in the brain. By clustering (or grouping) these responses one can reconstruct their underlying source, and also segregate it from other simultaneously interfering signals that are uncorrelated with it.

This simple principle of *temporal coherence* has already been shown to account experimentally for the perception of sources (or streams) in complex backgrounds [25]–[28]. However, this is the first detailed computational implementation of this idea that demonstrates how it works, and why it is so effective as a strategy to segregate spectrotemporally complex stimuli such as speech and music. Furthermore, it should be emphasized that despite apparent similarities, the idea of temporal coherence differs fundamentally from previous efforts that invoked correlations and synchronization in the following ways [29]–[33]: (1) coincidence here refers to that among modulated feature channels due to slow stimulus power (envelope) fluctuations, and not to any *intrinsic* brain oscillations; (2) coincidences are strictly done at cortical time-scales of a few hertz, and not at the fast pitch or acoustic frequency rates often considered; (3) coincidences are measured among modulated cortical features and perceptual attributes that usually occupy well-separated channels, unlike the crowded frequency channels of the auditory spectrogram; (4) coincidence must be measured over multiple time-scales and not just over a single time-window that is bound to be too long or too short for a subset of modulations; and finally (5) the details we describe later for how the coincidence matrices are exploited to segregate the sources are new and are critical for the success of this effort. For all these reasons, the simple principle of temporal coherence is not easily implementable. Our goal here is to show how to do so using plausible cortical mechanisms able to segregate realistic mixtures of complex signals.

As we shall demonstrate, the proposed framework mimics human and animal strategies to segregate sources with no prior information or knowledge of their properties. The model can also gracefully utilize available cognitive influences such as attention to, or memory of specific attributes of a source (e.g., its pitch or timbre) to segregate it from its background. We begin with a sketch of the model stages, with emphasis on the unique aspects critical for its function. We then explore how separation of feature channel responses and their temporal continuity contribute to source segregation, and the potential helpful role of perceptual attributes like pitch and location in this process. Finally, we extend the results to the segregation of complex natural signals such as speech mixtures, and speech in noise or music.

## Results

**Figure 1:**
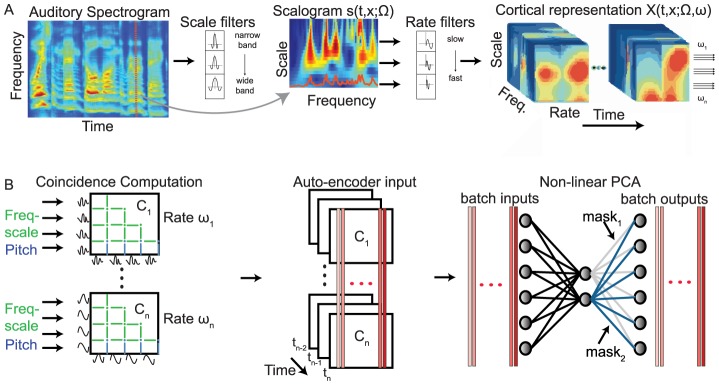
The temporal coherence model consists of two stages. (A) Transformation of sound into a cortical representation [34]: It begins with a computation of the auditory spectrogram (left panel), followed by an analysis of its spectral and temporal modulations in two steps (middle and right panels, respectively): a multi-scale (or a multi-bandwidth) wavelet analysis along the spectral dimension to create the frequency-scale responses, 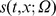, followed by a wavelet analysis of the *modulus* of these outputs to create the final cortical outputs 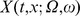 (right panel). (B) *Coincidence and clustering*: The cortical outputs at each time-step are used to compute a family of coincidence matrices (left panel). Each matrix (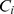) is the outer product of the cortical outputs 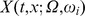 (i.e., separately for each modulation rate 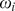). The C-matrices are then stacked (middle panel) and simultaneously decomposed by a nonlinear auto-encoder network (right panel) into two principal components corresponding to the foreground and background masks which are used to segregate the cortical response.

The temporal coherence algorithm consists of an auditory model that transforms the acoustic stimulus to its cortical representation (Fig. 1A). A subsequent stage computes a coincidence matrix (C-matrices in Fig. 1B) that summarizes the pair-wise coincidences (or correlations at zero-lag) between all pairs of responses making up the cortical representation. A final auto-encoder network is then used to decompose the coincidence matrix into its different streams. The use of the cortical representation here is extremely important as it provides a multiresolution view of the signal's spectral and temporal features, and these in turn endow the process with its robust character. Details of these auditory transformations are described elsewhere [34], and summarized in **Methods** below for completeness.

### Extracting streams from the coincidence matrices

The critical information for identifying the perceived sources is contained in the instantaneous coincidence among the feature channel pairs as depicted in the C-matrices (Fig. 1B). At each modulation rate 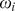, the coincidence matrix at time 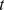 is computed by taking the outer product of all cortical frequency-scale 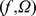 outputs (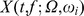). Such a computation effectively estimates simultaneously the "average coincidence" over the time window implicit in each 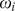 rate, i.e., at different temporal resolutions, thus retaining both short- and long-term coincidence measures crucial for segregation. Intuitively, the idea is that responses from pairs of channels that are strongly positively correlated should belong to the same stream, while channels that are uncorrelated or anti-correlated should belong to different streams. This decomposition need not be all-or-none, but rather responses of a given channel can be parceled to different streams in proportion to the degree of the average coincidence it exhibits with the two streams. This intuitive reasoning is captured by a factorization of the coincidence matrix into two uncorrelated streams by determining the direction of maximal incoherence between the incoming stimulus patterns. One such factorization algorithm is a nonlinear principal component analysis (nPCA) of the C-matrices [35], where the principal eigenvectors correspond to masks that select the channels that are positively correlated within a stream, and parcel out the others to a different stream. This procedure is implemented by an auto-encoder network with two rectifying linear hidden units corresponding to foreground and background streams as shown in Fig. 1B (right panel). The weights computed in the output branches of each unit are associated with each of the two sources in the input mixture, and the number of hidden units can be automatically increased if more than two segregated streams are anticipated. The nPCA is preferred over a linear PCA because the former assigns the channels of the two (often anti-correlated) sources to different eigenvectors, instead of combining them on opposite directions of a single eigenvector [36].

Another key innovation in the model implementation is that the nPCA decomposition is performed not directly on the input data from the cortical model (which are modulated at 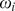 rates), but rather on the columns of the C-matrices whose entries are either stationary or vary slowly regardless of the 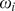 rates of the coincident channels. These common and slow dynamics enables stacking *all* C-matrices into one large matrix decomposition (Fig. 1B). Specifically, the columns of the stacked matrices are applied (as a batch) to the auto-encoder network at each instant 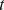 with the aim of computing weights that can reconstruct them while minimizing the mean-square reconstruction error. Linking these matrices has two critical advantages: It ensures that the pair of eigenvectors from each matrix decomposition is consistently labeled across all matrices (e.g., source 1 is associated with eigenvector 1 in all matrices); It also couples the eigenvectors and balances their contributions to the minimization of the MSE in the auto-encoder. The weight vectors thus computed are then applied as masks on the cortical outputs 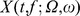. This procedure is repeated at each time step as the coincidence matrices evolve with the changing inputs.

### Role of feature separation, temporal continuity, and pitch in source segregation

The separation of feature responses on different channels and their temporal continuity are two important properties of the model that allow temporal coherence to segregate sources. Several additional perceptual attributes can play a significant role including pitch, spatial location, and timbre. Here we shall focus on pitch as an example of such attributes.

#### Feature separation

**Figure 2:**
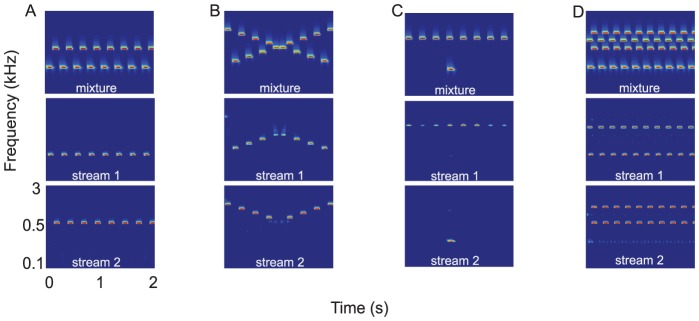
Stream segregation of tone sequences and complexes. Top row of panels represent the "mixture" audio whose two segregated streams are depicted in the middle and bottom rows. (A) The classic case of the well-separated alternating tones (top panel) becoming rapidly segregated into two streams (middle and bottom panels). (B) Continuity of the streams causes the crossing alternating tone sequences (top) to bounce maintaining an upper and a lower stream (middle and bottom panels). (C) Continuity also helps a stream maintain its integrity despite a transient synchronization with another tone. (D) When a sequence of tone complexes becomes desynchronized by more than 40 ms (top panel), they segregate into different streams despite a significant overlap (middle and bottom panels).

This refers to the notion that for two sounds to be segregated, it is necessary (but insufficient) that their features induce responses in mostly different auditory channels. Temporal coherence then serves to bind the coincident channels and segregate them as one source. For example, the tone sequences of Fig. 2A, B are well separated at the start, and are alternating and hence non-coincident. The sequences therefore quickly stream apart perceptually and become two segregated streams of high and low tones [1]. When the tones approach each other and their responses interact (as in Fig. 2B), the channels become more coherent and the segregation fails, as is evident by the middle tones becoming momentarily attenuated in the two segregated sequences [25].

#### Temporal continuity

The relatively slow dynamics of the cortical rate-filters (tuned at 2–16 Hz) confer this important property on streams. Specifically, the C-matrix entries inherit the dynamics of their rate-filters and hence change only as fast as the rate of their inputs, exhibiting an *inertia* or continuity. This explains why a tone sequence of rapidly alternating tones across two frequency channels splits into two streams each composed of slowly changing or stationary tones. By contrast, when a tone sequence changes its frequencies slowly, a stream can track the slow change and maintain the ongoing organization (as demonstrated by the slowly varying upper and lower frequency streams of the “bouncing-tone" sequence in Fig. 2B). Another example is when a new distant-frequency tone suddenly appears in a sequence, the C-matrix entries cannot track it rapidly enough causing the sequence to segregate and form a new stream that perceptually pops-out of the ongoing background (Fig. 2C). Finally, the bandpass character of cortical rate-filtering enhances the response to tone onsets (relative to their sustained portions), and hence repeated desynchronization of *onsets* is sufficient to segregate tone sequences despite extensive overlap as seen in Fig. 2D. These same phenomena are commonly seen with mixtures of more complex signals such as speech and music where the continuity of different streams is maintained despite transient synchronization and overlap.

#### How pitch contributes to segregation

**Figure 3:**
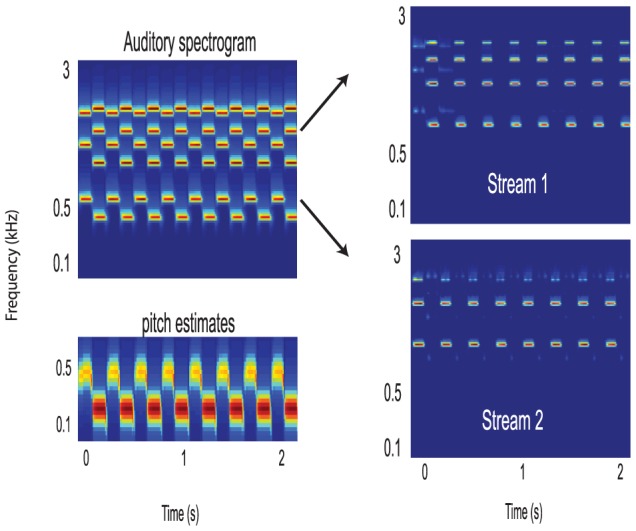
Segregation of harmonic complexes by the temporal coherence model. (A) A sequence of alternating harmonic complexes (pitches  = 500 and 630 Hz). (B) The complexes are segregated using all spectral and pitch channels. Closely spaced harmonics (1890, 2000 Hz) mutually interact and hence their channels are only partially correlated with the remaining harmonics, becoming weak or may even vanish in the segregated streams.

Harmonic complexes evoke pitch percepts at their fundamental and are commonly found in speech and music (see **Methods** for details). Fig. 3A illustrates how two such alternating complexes with different pitches (500 Hz and 630 Hz) form two streams. Aside from the spectral channels, we also plot the pitch of the complexes alternating below the spectrograms. The pitch estimates are computed with a harmonic-template algorithm [37], and mapped to an array of channels tuned to different values (see **Methods** for details), e.g., as in the pitch-selective neurons reported in the inferior colliculus or the auditory cortex [38], [39]. We refer to the activity of this pitch-ordered array of channels as a pitch-gram. These pitch channels are exploited in the coincidence matrix computations in an analogous way to the channels of the auditory spectrograms. That is, they are simply augmented to the spectral channels to create a larger feature vector that is used to compute a correspondingly larger coincidence matrix. The additional pitch channels contribute to the segregation of the alternating complexes of Fig.3A. Thus, despite having some closely spaced harmonics (1890, 2000 Hz), the two complexes are sufficiently different in pitch (and in other spectral components) that they produce largely uncorrelated responses in their pitch and spectral channels and hence can be readily segregated. The C-matrices in this simulation utilize all spectral and pitch channels. Note however, that not all these channels are necessary as comparable segregation can be achieved based only on a subset of channels. For example, since the pitch channel responses are correlated with their own spectral harmonics, it is sufficient to compute the nPCA decomposition only on the columns of the pitch channels in the C-matrices (see **Methods** for more details) to segregate the two complex sequences. Similarly, using coincidences between spectral scale-frequency inputs alone also yields similar segregation. In fact, if the pitch range of one harmonic complex is known (e.g., the pitch of the first complex is in the range 450 to 550 Hz), then its stream can be readily extracted by iterating the auto-encoder on the columns of the C-matrix that lie *only* in this pitch range. All these variations illustrate that the C-matrices can be exploited in various ways to segregate sources depending on availability of the different sound attributes, and that even partial information is often sufficient to form the streams and bind all their correlated components together. For example, if the location information is extracted and is available to the C-matrices (analogous to the pitch-grams), then they can be exploited in parallel with, and in a manner exactly analogous to the pitch. Temporal coherence can similarly help segregate speech using co-modulated signals of other modalities as in lip-reading as demonstrated later.

### Segregating speech from mixtures

**Figure 4:**
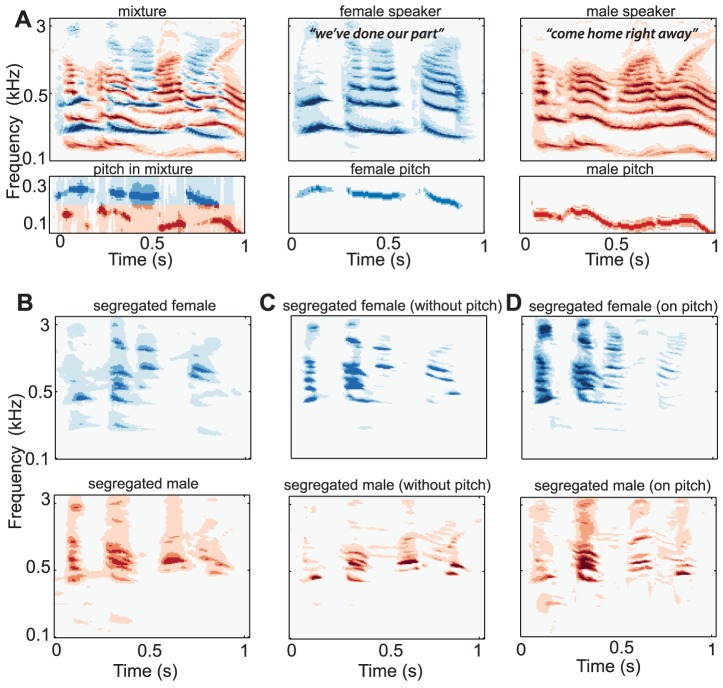
Segregation of speech mixtures. (A) Mixture of two sample utterances (left panel) spoken by a female (middle panel) and male (right panel); pitch tracks of the utterances are shown below each panel. (B) The segregated speech using all C-matrix columns. (C) The segregated speech using only coincidences among the frequency-scale channels (*no pitch* information). (D) The segregated speech using the channels surrounding the pitch channels of the female speaker as the anchor.

Speech mixtures share many of the same characteristics already seen in the examples of Fig. 2 and Fig. 3. For instance, they contain harmonic complexes with different pitches (e.g., males versus females) that often have closely spaced or temporally overlapped components. Speech also possesses other features such as broad bursts of noise immediately followed or preceded by voiced segments (as in various consonant-vowel combinations), or even accompanied by voicing (voiced consonants and fricatives). In all these cases, the syllabic onsets of one speaker synchronize a host of channels driven by the harmonics of the voicing, and that are desynchronized (or uncorrelated) with the channels driven by the other speaker. Fig. 4A depicts the clean spectra of two speech utterances (middle and right panels) and their mixture (left panel) illustrating the harmonic spectra and the temporal fluctuations in the speech signal at 3–7 Hz that make speech resemble the earlier harmonic sequences. The pitch tracks associated with each of these panels are shown below them.

Fig. 4B illustrates the segregation of the two speech streams from the mixture using all available coincidence among the spectral (frequency-scale) and pitch channels in the C-matrices. The reconstructed spectrograms are not identical to the originals (Fig. 4A), an inevitable consequence of the energetic masking among the crisscrossing components of the two speakers. Nevertheless, with two speakers there are sufficient gaps between the syllables of each speaker to provide clean, unmasked views of the other speaker's signal [40]. If more speakers are added to the mix, such gaps become sparser and the amount of energetic masking increases, and that is why it is harder to segregate one speaker in a crowd if they are not distinguished by unique features or a louder signal. An interesting aspect of speech is that the relative amplitudes of its harmonics vary widely over time reflecting the changing formants of different phonemes. Consequently, the saliency of the harmonic components changes continually, with weaker ones dropping out of the mixture as they become completely masked by the stronger components. Despite these changes, speech syllables of one speaker maintain a stable representation of a sufficient number of features from one time instant to the next, and thus can maintain the continuity of their stream. This is especially true of the pitch (which changes only slowly and relatively little during normal speech). The same is true of the spectral region of maximum energy which reflects the average formant locations of a given speaker, reflecting partially the timbre and length of their vocal tract. Humans utilize either of these cues alone or in conjunction with additional cues to segregate mixtures. For instance, to segregate speech with overlapping pitch ranges (a mixture of male speakers), one may rely on the different spectral envelopes (timbres), or on other potentially different features such as location or loudness. Humans can also exploit more complex factors such as higher-level linguistic knowledge and memory as we discuss later.

In the example of Fig. 4C, the two speakers of Fig. 4A are segregated based on the coincidence of only the spectral components conveyed by the frequency-scale channels. The extracted speech streams of the two speakers resemble the original unmixed signals, and their reconstructions exhibit significantly less mutual interference than the mixture as quantified later.Finally, as we discuss in more detail below, it is possible to segregate the speech mixture based on the pattern of correlations computed with one “anchor” feature such as the pitch channels of the female, i.e., using only the columns of the C-matrix near the female pitch channels as illustrated in Fig. 4D.

**Figure 5:**
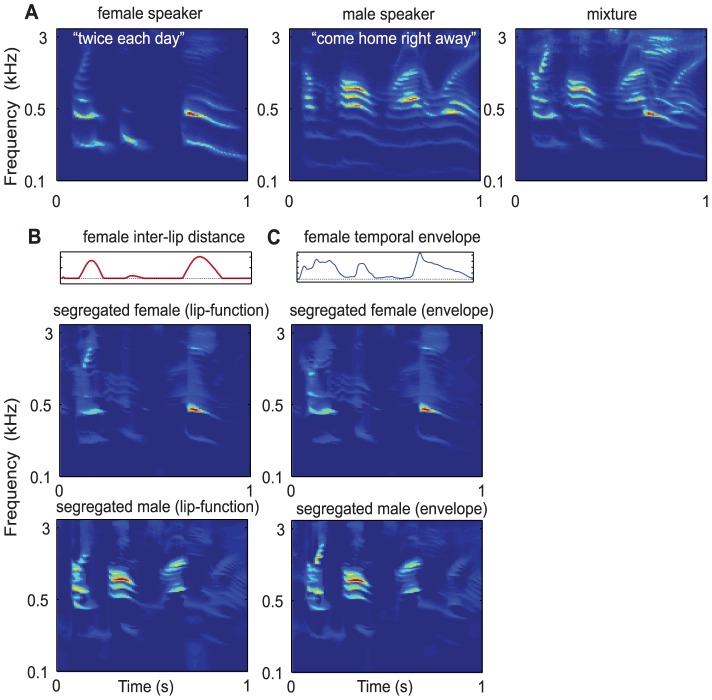
Segregation of speech utterances based on auxiliary functions. (A) Mixture of two sample utterances (right panel) spoken by a female (left panel) and male (middle panel) speakers; (B) The inter-lip distance of the female saying *“twice each day”* used as the anchor to segregate the mixture into its target female (middle panel) and the remaining male speech (bottom panel); (C) The envelope of the female speech “twice each day” used as anchor to segregate the mixture into its target female speaker (middle panel) and the remaining male speech (bottom speech).

Exactly the same logic can be applied to any auxiliary function that is co-modulated in the same manner as the rest of the speech signal. For instance, one may “look” at the lip movements of a speaker which open and close in a manner that closely reflects the instantaneous power in the signal (or its envelope) as demonstrated in [41]. These two functions (inter-lip distance and the acoustic envelope) can then be exploited to segregate the target speech much as with the pitch channels earlier. Thus, by simply computing the correlation between the lip function (Fig. 5B) or the acoustic envelope (Fig. 5C) with all the remaining channels, an effective mask can be readily computed to extract the target female speech (and the background male speech too). This example thus illustrates how in general any other co-modulated features of the speech signal (e.g., location, loudness, timbre, and visual signals such as lip movements can contribute to segregation of complex mixtures).

**Figure 6:**
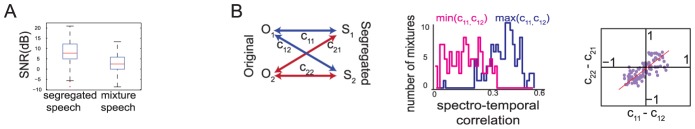
Signal to noise ratio. (**A**) Box plot of the SNR of the segregated speech and the mixture over 100 mixtures from the TIMIT corpus. (**B**) (Top) Notation used for coincidence measures computed between the original and segregated sentences plotted in panels below. (Middle) Distribution of coincidence in the cortical domain between each segregated speech and its corresponding original version (violet) and original interferer (magenta). 100 pairs of sentences from the TIMIT corpus were mixed together with equal power. (Bottom) Scatter plot of difference between correlation of original sentences with each segregated sentence demonstrates that the two segregated sentences correlate well with different original sentences.

The performance of the model is quantified with a database of 100 mixtures formed from pairs of male-female speech randomly sampled from the TIMIT database (Fig. 6) where the spectra of the clean speech are compared to those of the corresponding segregated versions. The signal-to-noise ratio is computed as
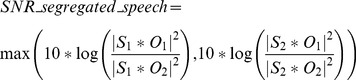
(1)

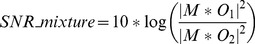
(2)


where 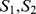 are the cortical representations of the segregated sentences and 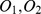 are the cortical representations of the original sentences and 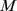 is the cortical representation of the mixture. Average SNR improvement was 6 dB for mixture waveforms mixed at 0 dB.

Another way to demonstrate the effectiveness of the segregation is to compare the match between the segregated samples and their corresponding originals. This is evidenced by the minimal overlap in Fig. 6B (middle panel) across the distributions of the coincidences computed between each segregated sentence and its original version versus the interfering speech. To compare directly these coincidences for each pair of mixed sentences, the difference between coincidences in each mixture are scatter-plotted in the bottom panel. Effective pairwise segregation (e.g., not extracting only one of the mixed sentences) places the scatter points along the diagonal. Examples of segregated and reconstructed audio files can be found in **S1 Dataset**.

#### Segregating speech from music and noise

**Figure 7:**
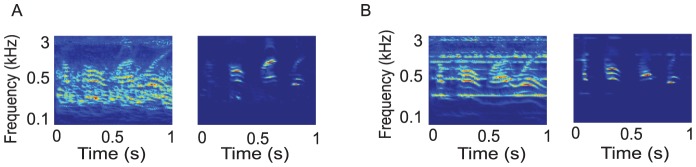
Extraction of speech from noise and music. (A) Speech mixed with street noise of many overlapping spectral peaks (left panel). The two signals are uncorrelated and hence can be readily segregated and the speech reconstructed (right panel). (B) Extraction of speech (right panel) from a mixture of speech and a sustained oboe melody (left panel).

In principle, segregating mixtures does not depend on them being speech or music, but rather that the signals have different spectrotemporal patterns and exhibit a continuity of features. Fig. 7A illustrates the extraction of a speech signal from a highly overlapping temporally modulated street noise background. The same speech sample is extracted from a mixture with music in Fig. 7B. As explained earlier, this segregation (psychoacoustically and in the model) becomes more challenging in the absence of “clean looks”, as when the background is an unmodulated white noise or babble that energetically masks the target speech.

### Attention and memory in streaming

So far, attention and memory have played no direct role in the segregation, but adding them is relatively straightforward. From a computational point of view, attention can be interpreted as a focus directed to one or a few features or feature subspaces of the cortical model which enhances their amplitudes relative to other unattended features. For instance, in segregating speech mixtures, one might choose to attend specifically to the high female pitch in a group of male speakers (Fig. 4D), or to attend to the location cues or the lip movements (Fig. 5C) and rely on them to segregate the speakers. In these cases, only the appropriate subset of columns of the C-matrices are needed to compute the nPCA decomposition (Fig. 1B). This is in fact also the interpretation of the simulations discussed in Fig. 3 for harmonic complexes. In all these cases, the segregation exploited only the C-matrix columns marking coincidences of the attended *anchor* channels (pitch, lip, loudness) with the remaining channels.

Memory can also be strongly implicated in stream segregation in that it constitutes *priors* about the sources which can be effectively utilized to process the C-matrices and perform the segregation. For example, in extracting the melody of the violins in a large orchestra, it is necessary to know first what the timbre of a violin is before one can turn the attentional focus to its unique spectral shape features and pitch range. One conceptually simple way (among many) of exploiting such information is to use as ‘template’ the average auto-encoder weights (masks) computed from iterating on clean patterns of a particular voice or instrument, and use the resulting weights to perform an initial segregation of the desired source by applying the mixture to the stored mask directly.

## Discussion

A biologically plausible model of auditory cortical processing can be used to implement the perceptual organization of auditory scenes into distinct auditory objects (streams). Two key ingredients are essential: (1) a multidimensional cortical representation of sound that explicitly encodes various acoustic features along which streaming can be induced; (2) clustering of the temporally coherent features into different streams. Temporal coherence is quantified by the coincidence between all pairs of cortical channels, slowly integrated at cortical time-scales as described in Fig. 1. An auto-encoder network mimicking Hebbian synaptic rules implements the clustering through nonlinear PCA to segregate the sound mixture into a foreground and a background.

The temporal coherence model segregates novel sounds based exclusively on the ongoing temporal coherence of their perceptual attributes. Previous efforts at exploiting explicitly or implicitly the correlations among stimulus features differed fundamentally in the details of their implementation. For example, some algorithms attempted to decompose directly the channels of the spectrogram representations [42] rather than the more distributed multi-scale cortical representations. They either used the fast phase-locked responses available in the early auditory system [43], or relied exclusively on the pitch-rate responses induced by interactions among the unresolved harmonics of a voiced sound [44]. Both these temporal cues, however, are much faster than cortical dynamics (>100 Hz) and are highly volatile to the phase-shifts induced in different spectral regions by mildly reverberant environments. The cortical model instead naturally exploits multi-scale dynamics and spectral analyses to define the structure of all these computations as well as their parameters. For instance, the product of the wavelet coefficients (entries of the C-matrices) naturally compute the running-coincidence between the channel pairs, integrated over a time-interval determined by the time-constants of the cortical rate-filters (Fig. 1 and **Methods**). This insures that all coincidences are integrated over time intervals that are commensurate with the dynamics of the underlying signals and that a balanced range of these windows are included to process slowly varying (2 Hz) up to rapidly changing (16 Hz) features.

The biological plausibility of this model rests on physiological and anatomical support for the two postulates of the model: a cortical multidimensional representation of sound and coherence-dependent computations. The cortical representation is the end-result of a sequence of transformations in the early and central auditory system with experimental support discussed in detail in [34]. The version used here incorporates only a frequency (tonotopic) axis, spectrotemporal analysis (scales and rates), and pitch analysis [37]. However, other features that are pre-cortically extracted can be readily added as inputs to the model such as spatial location (from interaural differences and elevation cues) and pitch of unresolved harmonics [45].

The second postulate concerns the crucial role of temporal coherence in streaming. It is a relatively recent hypothesis and hence direct tests remain scant. Nevertheless, targeted psychoacoustic studies have already provided perceptual support of the idea that coherence of stimulus-features is *necessary* for perception of streams [27], [28], [46], [47]. Parallel physiological experiments have also demonstrated that coherence is a critical ingredient in streaming and have provided indirect evidence of its mechanisms through rapidly adapting cooperative and competitive interactions between coherent and incoherent responses [26], [48]. Nevertheless, much more remains uncertain. For instance, where are these computations performed? How exactly are the (auto-encoder) clustering analyses implemented? And what exactly is the role of attentive listening (versus pre-attentive processing) in facilitating the various computations? All these uncertainties, however, invoke coincidence-based computations and adaptive mechanisms that have been widely studied or postulated such as coincidence detection and Hebbian associations [49], [50].

Dimensionality-reduction of the coincidence matrix (through nonlinear PCA) allows us effectively to cluster all correlated channels apart from others, thus grouping and designating them as belonging to distinct sources. This view bears a close relationship to the predictive clustering-based algorithm by [51] in which input feature vectors are gradually clustered (or routed) into distinct streams. In both the coherence and clustering algorithms, cortical dynamics play a crucial role in integrating incoming data into the appropriate streams, and therefore are expected to exhibit for the most part similar results. In some sense, the distinction between the two approaches is one of implementation rather than fundamental concepts. Clustering patterns and reducing their features are often (but not always) two sides of the same coin, and can be shown under certain conditions to be largely equivalent and yield similar clusters [52]. Nevertheless, from a biological perspective, it is important to adopt the correlation view as it suggests concrete mechanisms to explore.

Our emphasis thus far has been on demonstrating the ability of the model to perform unsupervised (automatic) source segregation, much like a listener that has no specific objectives. In reality, of course, humans and animals utilize intentions and attention to selectively segregate one source as the foreground against the remaining background. This operational mode would similarly apply in applications in which the user of a technology identifies a target voice to enhance and isolate from among several based on the pitch, timbre, location, or other attributes. The temporal coherence algorithm can be readily and gracefully adapted to incorporate such information and task objectives, as when specific subsets of the C-matrix columns are used to segregate a targeted stream (e.g., Fig. 3 and Fig. 4). In fact, our experience with the model suggests that segregation is usually of better quality and faster to compute with attentional priors.

In summary, we have described a model for segregating complex sound mixtures based on the temporal coherence principle. The model computes the coincidence of multi-scale cortical features and clusters the coherent responses as emanating from one source. It requires no prior information, statistics, or knowledge of source properties, but can gracefully incorporate them along with cognitive influences such as attention to, or memory of specific attributes of a target source to segregate it from its background. The model provides a testable framework of the physiological bases and psychophysical manifestations of this remarkable ability. Finally, the relevance of these ideas transcends the auditory modality to elucidate the robust visual perception of cluttered scenes [53], [54].

## Methods

### The auditory representation

Sound is first transformed into its auditory spectrogram, followed by a cortical spectrotemporal analysis of the modulations of the spectrogram (Fig. 1A) [34]. *Pitch* is an additional perceptual attribute that is derived from the resolved (low-order) harmonics and used in the model [37]. It is represented as a ‘pitch-gram’ of additional channels that are simply augmented to the cortical spectral channels prior to subsequent rate analysis (see below). Other perceptual attributes such as location and unresolved harmonic pitch can also be computed and represented by an array of channels analogously to the pitch estimates.

The auditory spectrogram, denoted by 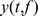, is generated by a model of early auditory processing [55], which begins with an affine wavelet transform of the acoustic signal, followed by nonlinear rectification and compression, and lateral inhibition to sharpen features. This results in *F* = 128 frequency channels that are equally spaced on a logarithmic frequency axis over 5.2 octaves.

Cortical spectro-temporal analysis of the spectrogram is effectively performed in two steps [34]: a spectral wavelet decomposition followed by a temporal wavelet decomposition, as depicted in Fig. 1A. The first analysis provides multi-scale (multi-bandwidth) views of each spectral slice 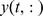, resulting in a 2D *frequency-scale* representation 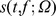. It is implemented by convolving the spectral slice with 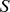 complex-valued spectral receptive fields 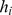 similar to Gabor functions, parametrized by spectral tuning 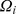, i.e., 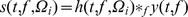.

The outcome of this step is an array of *F*x*S* frequency-scale channels indexed by frequency 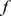 and local spectral bandwidth 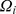 at each time instant *t*. We typically used 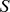  = 2 to 5 scales in our simulations (e.g., 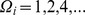 cyc/oct), producing 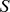 copies of the spectrogram channels with different degrees of spectral smoothing. In addition, the pitch of each spectrogram frame is also computed (if desired) using a harmonic template-matching algorithm [37]. Pitch values and saliency were then expressed as a *pitch-gram* (*P*) channels that are appended to the frequency-scale channels (Fig. 1B).

The cortical rate-analysis is then applied to the modulus of each of the channel outputs in the freq-scale-pitch array by passing them through an array 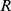 of modulation-selective filters (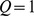), each indexed by its center rate 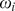 which range over 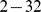 Hz in 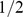 octave steps (Fig. 1B). This temporal wavelet analysis of the response of each channel is described in detail in [34]. Therefore, the final representation of the cortical outputs (features) is along four axes denoted by 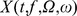. It consists of 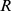 coincidence matrices per time frame, each of size 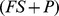
x(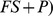 (Fig. 1B).

The exact choice of all above parameters is not critical for the model in that the performance changes very gradually when the parameters or number of feature channels are altered. All parameter values in the model were chosen based on previous simulations with the various components of the model. For example, the choice of rates (2–32 Hz) and scales (1–8 cyc/oct) reflected their utility in the representation of speech and other complex sounds in numerous previous applications of the cortical model [34]. Thus, the parameters chosen were known to reflect speech and music, but ofcourse could have been chosen differently if the stimuli were drastically different. The least committal choice is to include the largest range of scales and rates that is computationally feasible. In our implementations, the algorithm became noticeably slow when 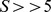, 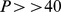, 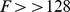, and 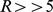.

### Coherence computations and nonlinear principal component analysis

The decomposition of the C-matrices is carried out as described earlier in Fig. 1B. The iterative procedure to learn the auto-encoder weights employs Limited-memory Broyden-Fletcher-Goldfarb-Shannon (L-BFGS) method as implemented in [56]. The *output* weight vectors (Fig. 1B) thus computed are subsequently applied as masks on the input channels 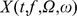. This procedure that is repeated every time step using the weights learned in the previous time step as initial conditions to ensure that the assignment of the learned eigenvectors remains consistent over time. Note that the C matrices do not change rapidly, but rather slowly, as fast as the time-constants of their corresponding rate analyses allow (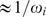). For example, for the 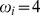 Hz filters, the cortical outputs change slowly reflecting a time-constant of approximately 250 ms. More often, however, the C-matrix entries change much slower reflecting the sustained coincidence patterns between different channels. For example, in the simple case of two alternating tones (Fig. 2A), the C-matrix entries reach a steady state after a fraction of a second, and then remain constant reflecting the unchanging coincidence pattern between the two tones. Similarly, if the pitch of a speaker remains relatively constant, then the correlation between the harmonic channels remains approximately constant since the partials are modulated similarly in time. This aspect of the model explains the source of the continuity in the streams. The final step in the model is to invert the *masked* cortical outputs 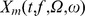 back to the sound [34].

## Supporting Information

S1 Dataset **Example segregation of a male-female mixture.** The female sentence is *‘The clothes dried on a thin wooden rack’*. The male sentence is *‘The juice of lemons makes fine punch’*. *Female_original.wav* is the original female speech. *Male_original.wav* is the original male speech. *Mixture.wav* is the 0 dB mixture speech. *Female_reconstructed.wav* is the segregated female speech and *Male_reconstructed.wav* is the segregated male speech.
(ZIP)
